# Prediction of re-oxidation behaviour of ultra-low carbon steel by different slag series

**DOI:** 10.1038/s41598-020-66318-w

**Published:** 2020-06-10

**Authors:** Aijun Deng, Yunjin Xia, Hongbiao Dong, Haichuan Wang, Dingdong Fan

**Affiliations:** 1 School of Metallurgical Engineering, Anhui University of Technology, Ma’anshan, 243002 People’s Republic of China; 2 Department of Engineering, University of Leicester, Leicester, LE1 7RH United Kingdom

**Keywords:** Engineering, Materials science

## Abstract

A kinetic model was developed using FactSage Macro Processing to simulate the re-oxidation of ultra-low carbon steel via different oxidising slags. The calculated results show good agreement with experimental laboratory thermal simulation data. Therefore, the model can be used to predict the change behaviour of slag-metal-inclusion in the re-oxidation reaction of liquid steel. It can provide prediction and guidance for an accurate secondary oxidation control process. During the slag re-oxidation process, when the oxygen in the steel is supersaturated and the slag is low in oxidation, it can easily form stick-like and dendritic shape inclusions of Al_2_O_3_ in steel. As the (FeO) content increases in slag, the oxygen transfer from slag to steel is evident, and the inclusion size increases, showing clusters and spherical shapes. In addition, supersaturated oxygen in steel easily forms unstable Al_2_O_3_-TiO_x_ inclusions with [Ti]. As the components of liquid steel tend to be uniform, the Al_2_O_3_-TiO_x_ inclusions will decompose and disappear, forming stable Al_2_O_3_ and TiO_2_ inclusions. The number of inclusions can be reduced by increasing the basicity and the ratio of CaO to Al_2_O_3_ in the initial slag.

## Introduction

Ultra-low carbon steel is widely used in the production of automotive and home appliance panels. To ensure excellent ductility, deep drawability, and good surface quality of metal materials, it has high control requirements for the types, contents, and forms of inclusions in steel^1,2^. The process route for producing ultra-low carbon steel is Converter → Argon Blowing Station → Ruhstahl-Heraeus (RH) → Continuous Casting. During ladle processing, Al granules are added as a deoxidiser, and Ti is added as alloying treatment. Since the ladle top slag cannot be fully reduced in this process like it can in ladle furnace (LF) refining, after RH refining, the ladle top slag still has strong oxidising properties, and the (FeO) in the slag transmits oxygen to the steel. It reacts with elements such as Al and Ti in the steel, which forms Al_2_O_3_, TiO_x_, and Al-Ti-O composite inclusions. It affects the cleanliness of liquid steel, which can cause nozzle blockage in severe cases^3–5^. Therefore, the activity of (FeO) in the slag is reduced by modifying the top slag with high oxidation properties. Reducing this activity can effectively control the oxygen transfer to steel and reduce the T.[O] content in steel, thereby enhancing the purity of liquid steel and improving the casting process of liquid steel^6–9^.

Scientists^10^ at Baosteel have determined that controlling the *w*(TFe)<8% in the slag can effectively reduce the T.[O] content of liquid steel in tundish to less than 30 ppm. Lee, *et al*.^11^ found that when *w*(TFe)>1.5% in the slag, the oxidation rate of slag to liquid steel increases linearly with the increase of (FeO) content in the slag. When *w*(TFe) is constant, the oxidation rate decreases with the increase of *w*(CaO)/*w*(Al_2_O_3_) in the slag. Ren, *et al*.^12^ studied the oxidation reaction of slag to liquid steel and found that it occurs in the slag-metal interface and that increasing the *w*(CaO)/*w*(SiO_2_) in the slag can significantly inhibit the self-dissociation of (SiO_2_) in the slag that transfers oxygen to steel, while it can reduce the formation of inclusions in liquid steel. There have been many studies on the relationship between the oxidation of slag and the T.[O] content in liquid steel, but little research exists regarding methods that change the oxidising components of the slag in a manner that affects the types and forms of inclusions in steel. Therefore, it is necessary to conduct in-depth research on the re-oxidation behaviour of differing oxidising slag and liquid steel.

However, steel smelting is a complex process of heterogeneous reaction at high temperature. The interfacial contact reaction of slag-metal is usually instantaneous. Researchers are unable to control and modulate this interfacial contact reaction by direct intervention. Traditional research methods can only be described and explained by the classical interface reaction theory, but these methods were far from meeting the needs of industrial production. With the development of computer and big data technology, it has become a low-cost and efficient method to study the relatively complex metallurgical reaction process. Thus, The complex metallurgical reaction process that cannot be explained and analyzed before was expressed by big data and computational model, and it was analyzed and discussed. It is proved that the calculation model is more practical and accurate than the traditional metallurgical theory in predicting and analyzing a complex metallurgical reaction process. The method of model prediction has greatly improved the understanding of complex metallurgical reactions. It is of great practical significance for the researchers and operators to optimize the process parameters and improve production efficiency. Recently, some scholars^13–17^ proposed some kinetic models, based on thermodynamic theoretical calculation and fluid dynamics equations, which can successfully predict changes in slag, liquid steel, and inclusions at the slag-metal interface. However, these models also have problems, such as decreased prediction accuracy and incomplete prediction of actual production requirements, that accompany changes in thermodynamic data and complex slag systems^18–20^.

This study uses ultra-low carbon steel as the research object and develops a kinetic model based on laboratory thermal simulation data combined with FactSage 7.2 Macro Processing software. The model can more intuitively describe the re-oxidation behaviour of slag with different oxidisability than liquid steel and more accurately predict the evolution of slag, liquid steel, and inclusions during the slag–metal interface reaction. It can also formulate more accurate and reasonable control technology for liquid steel re-oxidation.

## Experimental methods and procedures

### Materials

A steel mill was used to produce ultra-low carbon steel. When the steel was RH outbound, five samples were taken from the same ladle, each approximately 100 g. The chemical composition of the samples is shown in Table 1. In this experiment, CaO, SiO_2_, FeO, and MnO were used to simulate the main components of the ladle top slag. Five groups of slag were prepared separately. Their mass fractions are shown in Table 2. These slag systems were prepared and mixed, then placed in a corundum crucible and calcined at 1000 °C for pre-melting before use in the experiments.

**Table 1:** Ultra-low carbon steel composition at the end of RH refining, wt.%.

C	Si	Mn	P	S	Als	N	Ti	T.[O] /10^−6^
0.002	0.002	0.35	0.005	0.005	0.058	0.002	0.038	20

**Table 2:** Slag composition, wt.%.

No.	CaO	SiO_2_	FeO	MnO	Basicity (R = *w*_CaO_/ *w*_SiO2_)
I	73	25	1	1	2.92
II	73	24	2	1	3.04
III	68	23	8	1	2.96
IV	63	21	15	1	3
V	69	15	15	1	4.6

### Experimental process

**Figure 1:**
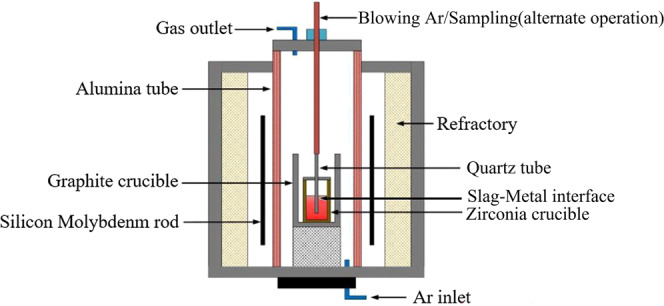
Schematic diagram of the experimental apparatus.

In this study, the constant basicity of the experimental I–IV slag was 3.0, and the MnO content was 1%. The experimental IV and V slag series use the same oxidisation and different basicity. The re-oxidation behaviour of the slag upon the liquid steel and the evolution of slag, liquid steel, and inclusions during the slag-metal reaction were studied by changing the oxidation (change in single-variable FeO content) and slag basicity. These changes provide thermodynamic experimental data for the establishment of a kinetic model. The schematic diagram of the experimental device is shown in Fig. 1. A silicon molybdenum furnace was used to provide a stable heat source for the whole experiment. The bottom of the furnace injects Ar gas throughout the experiment. The top of the furnace was sealed with a lid, and Ar gas was introduced into the whole furnace to isolate the influence of air on the experiment. The sampling tube on the top of the furnace can blow Ar gas to stir the reaction process, and stop blowing Ar gas at the end of the reaction time, which can be used as the sampling tube. This quartz tube can be alternately blown and sampled.

The slag-metal reaction was carried out at 1853 K. First, a 100 g sample was placed into the zirconia crucible and slowly heated to 1853 K. Then, 30 g pre-melted slag was added. The steel and slag samples were taken at the same time during the reaction at 5 min, 10 min, 15 min, 20 min, 25 min, and 30 min. Then, the above experimental procedure was repeated with a 100 g ingot and pre-melted slag of II, III, IV, and V, with samples taken in that order. The detailed experimental scheme and the sample numbers are shown in Table 3.

**Table 3:** Sample number with regard to different slag and reaction time.

Slag\Time	5 min	10 min	15 min	20 min	25 min	30 min
I	I-5	I-10	I-15	I-20	I-25	I-30
II	II-5	II-10	II-15	II-20	II-25	II-30
III	III-5	III-10	III-15	III-20	III-25	III-30
IV	IV-5	IV-10	IV-15	IV-20	IV-25	IV-30
V	V-5	V-10	V-15	V-20	V-25	V-30

### Sample preparation

**Figure 2:**
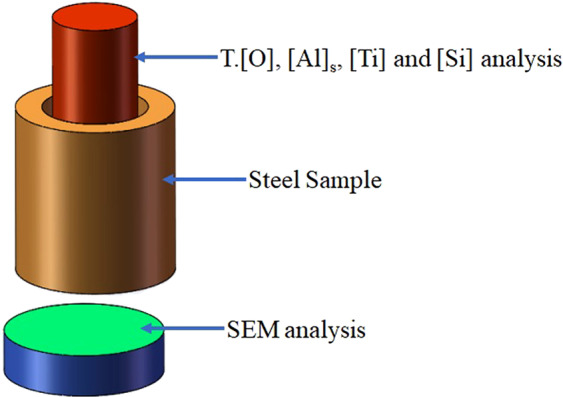
Schematic of the cut sample.

The experimental test analysis steel sample is shown in Fig. 2. The central part of the steel sample was processed into a φ5 × 40 mm steel rod. The total oxygen (T.[O]) content in the steel after the reaction was measured by an EMGA-620 oxygen-nitrogen detector, and the remaining elements were analysed by atomic emission spectrometry. Steel cakes of φ40 × 15 mm were cut from the bottom of the reaction steel samples from each group, and the inclusions were analysed using an ASPEX PSEM Explore scanning electron microscope, which can detect the number, size, and composition of inclusions in a specified area, in addition to performing conventional electron microscope functions. The composition, size, and area information for each inclusion was analysed using the analysis software of AZtecSteel. The slag composition of each stage of the experiment was obtained by X-ray fluorescence spectrometer analysis.

The detection area of all steel sample inclusions in this study was approximately 100 mm^2^. The typical characteristic inclusions in the scanning area were statistically analysed, and inclusions of the same type, shape, and size were not detected repeatedly.

## Results and Discussion

### Kinetic prediction of slag-steel-inclusion reaction during slag re-oxidation process

**Figure 3:**
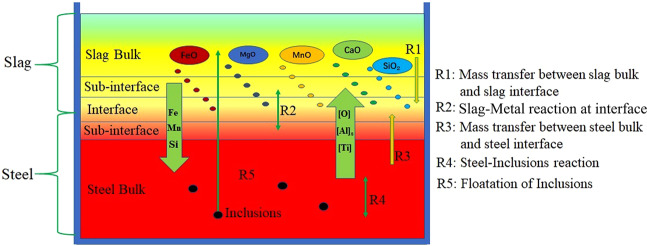
Schematic diagram of slag-metal-inclusions reaction during slag re-oxidation process.

The re-oxidation behaviour of liquid steel via slag in the ladle mainly involves the chemical reactions and processes as shown in Fig. 3. During the oxidation reaction, R1 and R3 determined the rate and direction of the slag-metal interface reaction. When the diffusion of [O] in steel is a limiting link, oxygen will diffuse from the slag to the liquid steel. At this time, the oxidation reaction R4 between oxygen and deoxidised elements such as [Al]_s_ and [Ti] in the steel occurs in inside of liquid steel. When the mass transfer of (SiO_2_), (FeO) and (MnO) in the slag phase is a limiting link, the oxidation reaction occurs in the slag-metal interface, that is, the R2 reaction, occurs. When the chemical potential of oxygen in the slag-metal phase is relatively close, the oxidation reaction is simultaneously restricted by mass transfer of [O] in steel and (SiO_2_), (FeO) and (MnO) in slag. The slag-metal interface is in equilibrium, and no obvious re-oxidation behaviour will occur at this time.

The accuracy and validity of the hypothesis conditions will be checked and corrected by the test data of the steel sample and slag sample. To facilitate the establishment and calculation of the model, the thermodynamic experiment and the kinetic model need to make the following assumptions:

(1) In the thermal simulation experiment, when the slag is added to the steel to participate in the reaction, the slag reaction step size is Δ*t* = 5% m_slag_^21^. (2) The slag-metal interface reaction can reach an equilibrium state. (3) The inclusions formed by the reaction are evenly distributed in the slag bulk and the metal bulk. (4) The inclusions in the steel can fully float and be adsorbed by slag. (5) The floating rate of inclusions is constant. (6) The thermal simulation experimental data used in the model ignores the reaction effects of slag to the crucible and liquid steel to the crucible.

In the FactSage software, the Macro Processing module was invoked to create a balanced reaction program. All input data for the model are based on the data from this thermal simulation experiment, and reference data are appropriately cited. The output data were all organised into data lists or Microsoft Excel tables, which were processed and plotted into corresponding charts at a later stage^22^. All the major chemical reactions in Fig. 3 were formed into small programs by the Macro Processing code. All thermodynamic reaction equilibrium phase diagrams were calculated using FactPS, FToxid, and FTmisc databases in FactSage software under adiabatic conditions^23^.

### Model validation

**Figure 4:**
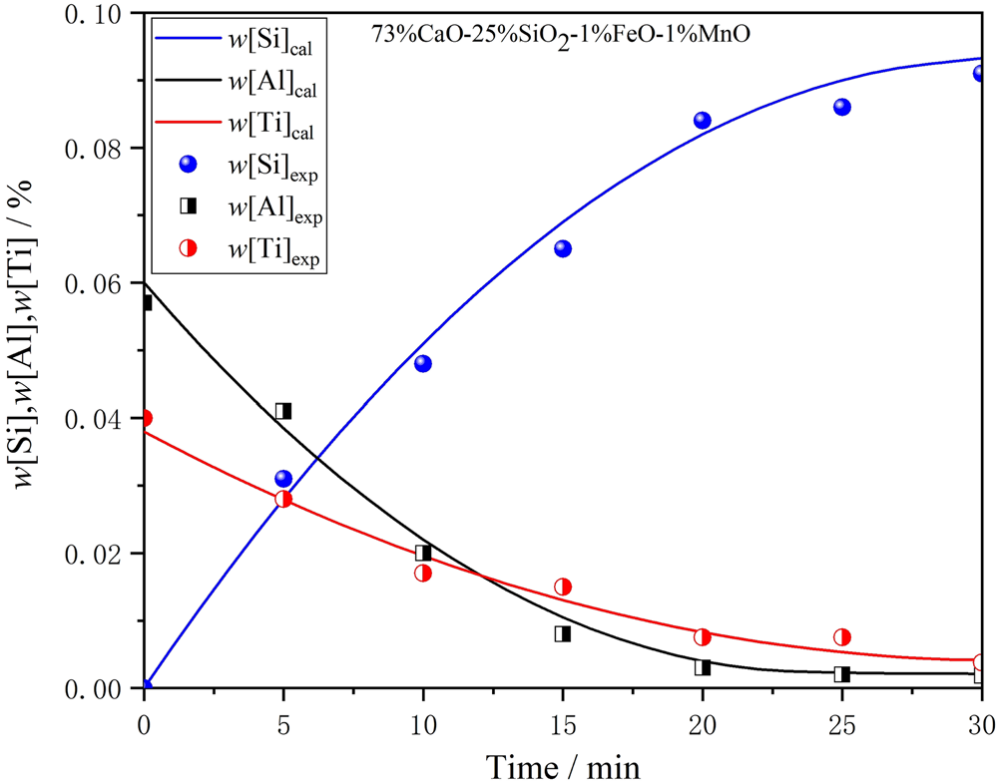
Measured *w*[Si]%, *w*[Al]%, and *w*[Ti]% from laboratory experiments compared with modeled value for *m*_steel_ = 1.25 × 10^−5^ m^3^/s.

To verify the accuracy of the model, the data adopted in this study were mainly based on the thermal simulation experiment, and a few references are cited. The basic conditions and data used in the reaction of slag I with ultra-low carbon steel were selected randomly as the input data for the kinetic model. The actual experimental value was compared with the output data calculated by the model, and the results are shown in Fig. 4. The inner diameter of the zirconia crucible used in the experiment was 60 mm, and the height was 80 mm. Thus, the reaction area of the interface is 0.0028 m^2^. The densities of slag and steel used in the calculation were, respectively, 2500 kg/m^3^ and 7200 kg/m^3^. The mass transfer coefficient of steel in this kinetic model can be estimated through the fitting experiment by measuring the concentration changes of [Al]_s_, [Ti], and [Si] in the steel, and repeated modifications were carried out to obtain the best fitting parameters. Figure 4 shows that when the estimated mass transfer coefficient of steel is 1.25 × 10^−5^ m^3^/s, the calculated results of this kinetic model show good consistency with the experimental values under the same conditions, which fully indicates that the mass transfer coefficient value can meet the calculation needs of the existing model.

The total oxygen (T.[O]) content in steel includes dissolved oxygen in liquid steel and oxygen in inclusions. When the slag oxidises the liquid steel, the chemical potential of the dissolved oxygen in steel and the oxide in slag tends to gradually balance, and the floating removal rate of the inclusions also has a significant impact on the oxygen in the inclusions.

**Figure 5:**
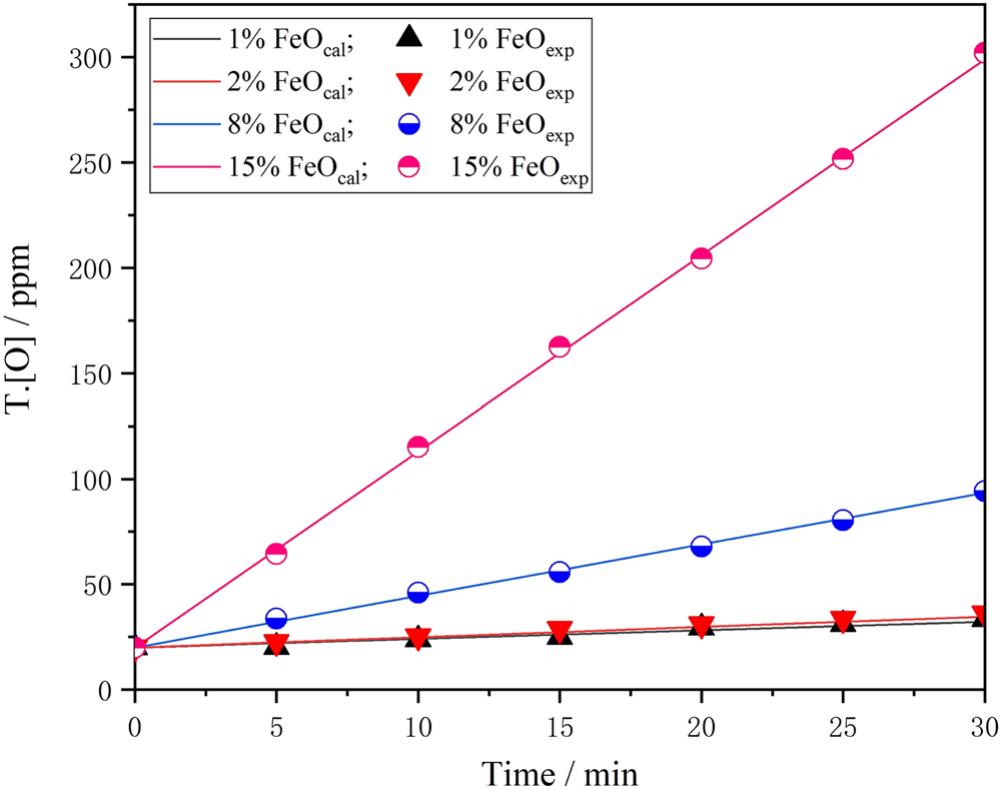
Measured T.[O] in steel from laboratory experiments compared with modeled value with various FeO content.

Figure 5 is a comparison of the change value in T.[O] content in the model calculation and the experimentally measured change value when different oxidising slag acts on the liquid steel. The figure shows that as the oxidising property of slag increases, the T.[O] content of steel calculated by the model increases linearly. Under the same conditions, the measured and calculated values show good consistency. This indicates that the kinetic model can better predict the change of T.[O] content in steel. Therefore, it can be considered that the kinetic model can meet actual production needs and provide prediction and guidance for the formulation of an accurate re-oxidation control process.

### Re-oxidation behaviour of ultra-low carbon steel by different oxidisability slags

To predict the re-oxidation behaviour of ultra-low carbon steel with differing oxidising slag, the process of oxidisation was simulated by FactSage Macro Processing. For the materials used for the model calculations, the steel was 100 g, and the five groups of different oxidising slags were 30 g each. The specific components of steel and slag systems are shown in Table 1 and Table 2, respectively. The calculated temperature was 1853 K.

When the slag interacts with ultra-low carbon steel, reactions (1–7) may occur in the slag-metal interface and the inside of liquid steel.

1 $$({{\rm{SiO}}}_{2})=[{\rm{Si}}]+2[{\rm{O}}]$$

2 $$({\rm{FeO}})=[{\rm{Fe}}]+[{\rm{O}}]$$

3 $$2{[{\rm{Al}}]}_{{\rm{s}}}+3[{\rm{O}}]={({{\rm{Al}}}_{2}{{\rm{O}}}_{3})}_{{\rm{inclusions}}}$$

4 $$2[{\rm{Ti}}]+3[{\rm{O}}]={({{\rm{Ti}}}_{2}{{\rm{O}}}_{3})}_{{\rm{inclusions}}}$$

5 $$[{\rm{Ti}}]+2[{\rm{O}}]={({{\rm{TiO}}}_{2})}_{{\rm{inclusions}}}$$

6 $${({{\rm{Ti}}}_{2}{{\rm{O}}}_{3})}_{{\rm{inclusions}}}+[{\rm{O}}]=2{({{\rm{TiO}}}_{2})}_{{\rm{inclusions}}}$$

7 $$2{[{\rm{Al}}]}_{{\rm{s}}}+[{\rm{Ti}}]+(3+{\rm{x}})[{\rm{O}}]={({{\rm{Al}}}_{2}{{\rm{O}}}_{3}-{{\rm{TiO}}}_{{\rm{x}}})}_{{\rm{inclusions}}}$$

**Figure 6:**
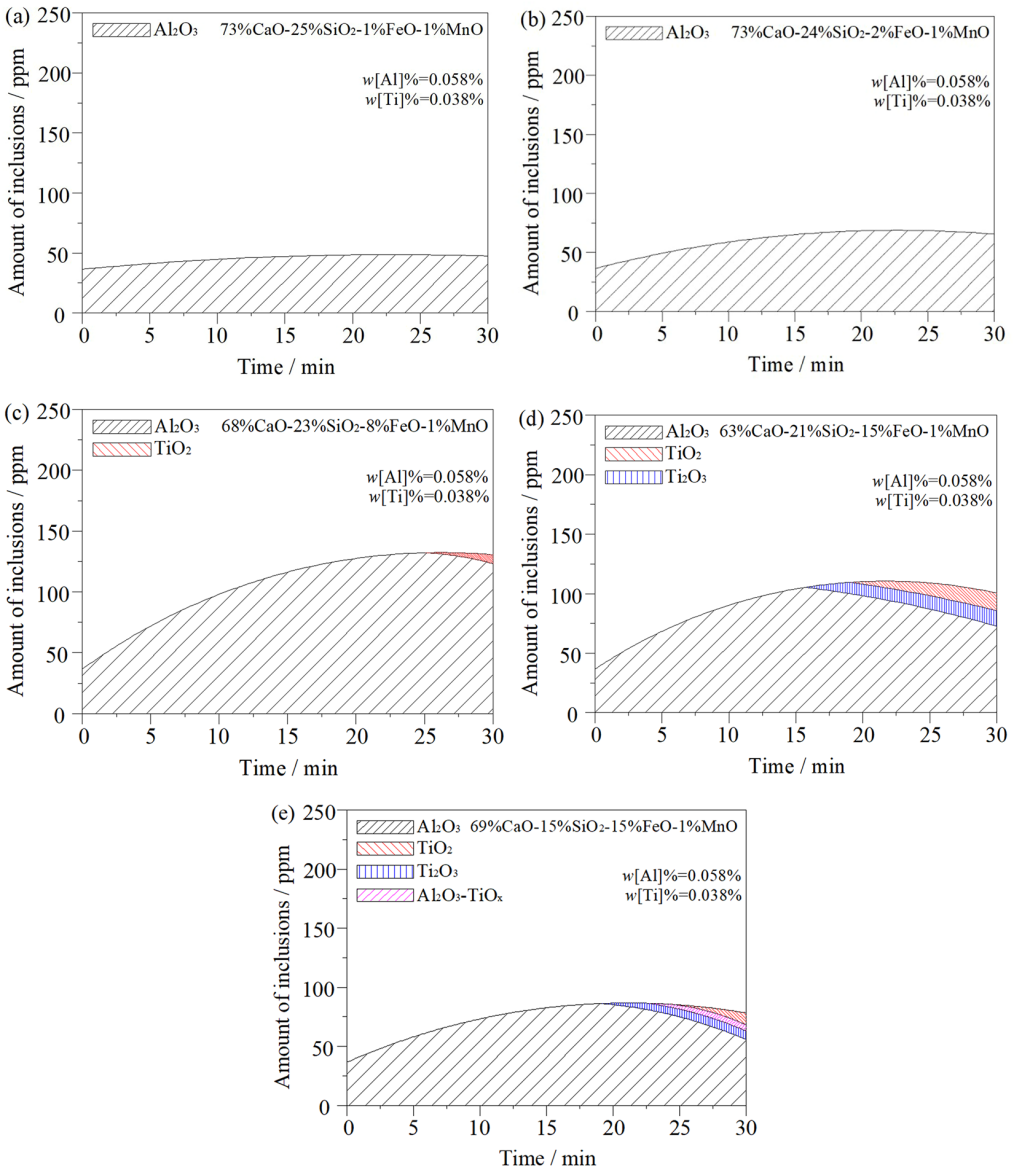
Effect of initial slag chemistry composition and basicity on change of inclusions in ultra-low carbon steel: (**a**) *w*(FeO)% = 1%, R = 3; (**b**) *w*(FeO)% = 2%, R = 3; (**c**) *w*(FeO)% = 8%, R = 3; (**d**) *w*(FeO)% = 15%, R = 3; (**e**) *w*(FeO)% = 15%, R = 4.6.

Figure 6 is a model for calculating the variation of inclusions in steel when different oxidising slags act on liquid steel. Combined with Fig. 5, the figures show that when *w*(FeO)% = 1%, the oxidation of slag is low. The T.[O] content increases slightly in liquid steel. The inclusions in steel are mainly Al_2_O_3_, and the total inclusions do not increase significantly with the change in reaction time, as shown in Fig. 6a. The model calculation shows that the oxygen generated by the inclusions mainly comes from the self-dissociation reaction of (SiO_2_) in the slag, and the [Si] content in steel increases significantly. In addition, because of the low (FeO) content in the slag, its activity is also lower. Therefore, under the influence of slag interfacial tension, there is no large-scale self-dissociation reaction to transfer oxygen to the steel^20^. Therefore, it can be inferred that the oxidation property of slag to liquid steel is mainly affected by (SiO_2_), but not by the (FeO) in slag. At this point, reactions (1) and (3) occur in the slag-metal interface. As the (FeO) content in the slag increases to 2%, the total number of inclusions in the steel increases slightly, as shown in Fig. 6b.

When *w*(FeO)% = 8% in the slag, the T.[O] content in the steel increases sharply. The oxygen transfer from slag to liquid steel is obvious, and the total inclusions in the steel increase correspondingly. Figure 6c indicates that when the reaction proceeds to approximately 25 minutes, TiO_2_ inclusions appear in the steel and Al_2_O_3_ inclusions decrease. During the whole reaction process, the oxidation of [Al]_s_ occurred in steel before [Ti] in the early stage of the reaction, and a large number of Al_2_O_3_ inclusions were formed. This is mainly because the binding force of aluminium to oxygen is much greater than that of titanium to oxygen. As the reaction proceeded, the [Al]_s_ content in the steel decreased in the later stages of the reaction, and some oxygen immediately oxidised the [Ti] to form TiO_2_ inclusions. According to the test results from the steel samples after the reaction, there was no significant change in [Si] content in the steel. Therefore, it can be inferred that the oxygen in steel mainly comes from the decomposition reaction of (FeO) in the slag. Moreover, its decomposition reaction inhibits the self-dissolution reaction of (SiO_2_) in the slag. The oxidation of slag mainly manifested as the (FeO) content in the slag, which is not directly related to the (SiO_2_) content in the slag. At this point, main reactions (2), (3), and (5) occur in the slag-metal interface and the inside of the liquid steel.

When *w*(FeO)% = 15%, as shown in Fig. 6d, Ti_2_O_3_ inclusions appear in the steel at the reaction time of 15 min, and TiO_2_ inclusions appear after another 5 min. With the continuous progress of the reaction, Al_2_O_3_ and Ti_2_O_3_ inclusions in the steel show a decreasing trend, while TiO_2_ inclusions show signs of increasing. This is mainly due to the poor stability of Ti_2_O_3_ at a high temperature; Ti_2_O_3_ easily combines with oxygen to form stable TiO_2_. At this point, main reactions (2), (3), (4), (5), and (6) occur in the slag–metal interface and the inside of the liquid steel. In addition, when Fig. 6d is compared with Fig. 6c, it can be seen that the total inclusions decrease throughout the whole reaction process. This is mainly due to the increase in Al_2_O_3_ inclusions generated in the early stage of the reaction to the slag, which improves the *w*(CaO)/*w*(Al_2_O_3_) in the slag, a condition conducive to improving the slag’s ability to absorb the inclusions.

Figure 6d,e show the re-oxidation behaviour of liquid steel via slag with the same oxidation properties but a different basicity. By comparing the calculation results in Fig. 6e,d, it can be seen that the basicity of slag has a great influence on the inclusions in steel. According to Fig. 6e, TiO_2_ and Ti_2_O_3_ inclusions began to appear in the steel at approximately 25 min into the late stage. The analysis shows that this is due to the increase in basicity. Meanwhile, the Al_2_O_3_ formed in the early stage of the reaction also increases the *w*(CaO)/*w*(Al_2_O_3_) of slag. These two aspects are conducive to inhibiting the premature oxidation of [Ti] in steel and avoiding the premature formation of Ti inclusions. The Al_2_O_3_-TiO_x_ solid solution phase appears in the steel at a later stage of the reaction. This is due to the decrease in [Al]_s_ content in steel in the later reaction stage and [Ti] may combine with a high concentration of oxygen. This forms a solid solution phase with TiO_x_ as the core and Al_2_O_3_ as the outer layer. This is consistent with the results in ref. ^24^.With the solidification of liquid steel, the Al_2_O_3_-TiO_x_ solid solution phase has no time to rise and remove, so it remains in the steel to form inclusions. Such inclusions are unstable and can be removed by raising the temperature of the liquid steel and increasing the duration of ladle bottom blowing. Main reactions (2), (3), (4), (5), (6), and (7) occur in the slag-metal interface and on the inside of the liquid steel. It can be seen from the comparison of Fig. 6d,e that with the increase in slag basicity, total inclusions in steel can be reduced, and the purity of liquid steel can be improved.

**Figure 7:**
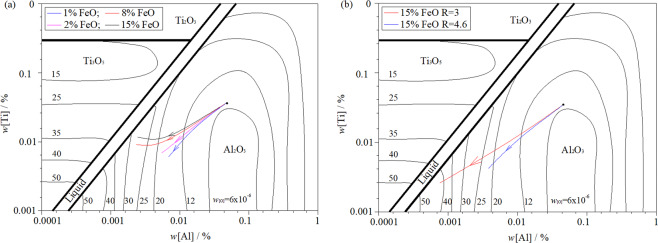
Effect of initial slag chemistry composition and basicity on change in [Al] and [Ti] in ultra-low carbon steel: (**a**) different chemical composition, same basicity; (**b**) different basicity, same chemical composition.

Figure 7 shows the stable phase diagram of Al-Ti-O inclusions formed in steel at 1853 K. This was calculated using the FactPs, FToxid, and FTmisc databases in FactSage 7.2. The arrow direction in the figure indicates the region change of stable inclusion formation when the content of [Al]_s_ and [Ti] in steel changes with the re-oxidation of liquid steel. As indicated in Fig. 7a, when *w*[Al]% = 0.058% and *w*[Ti]% = 0.038%, stable Al_2_O_3_ inclusions can be formed at the beginning of the oxidation reaction. With continuous reaction, the [Al]_s_ content in steel decreases continuously, and unstable Al_2_O_3_-TiO_x_ inclusions begin to form in the steel. With an oxidisation increase, Al_2_O_3_-TiO_x_ inclusions gradually transform into Ti_2_O_3_ or TiO_2_ in a stable state. This is consistent with the calculation in Fig. 6. As can be seen from Fig. 7b, the slag with the same oxidation properties. The basicity of the initial slag and the *w*(CaO)/*w*(Al_2_O_3_) of the slag also increases with the re-oxidation reaction. It can significantly suppress the oxidation of the slag into [Ti] and reduce the formation of Ti inclusions in the steel.

### Evolution of inclusions in steel during slag re-oxidation process

**Figure 8:**
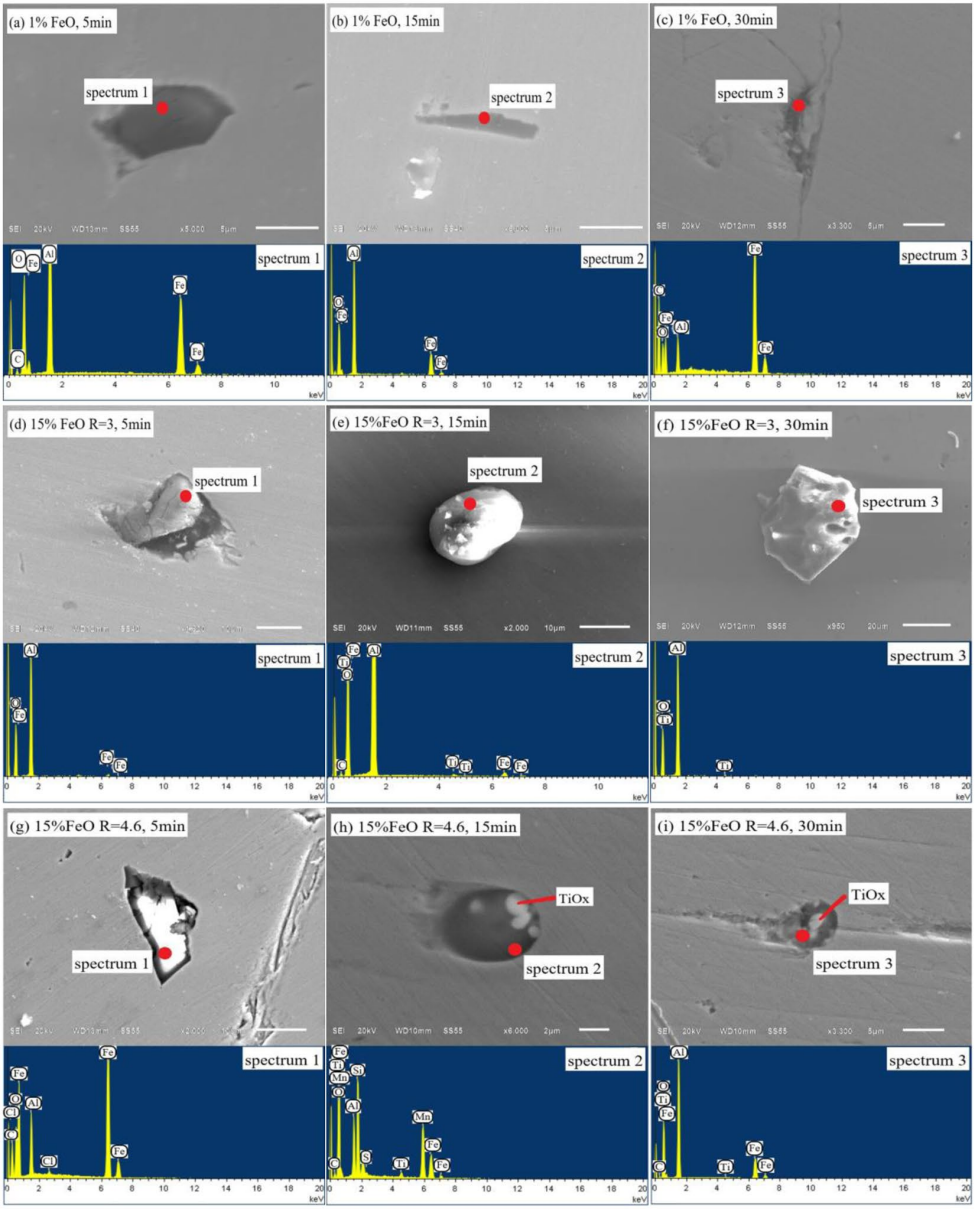
Evolution of inclusions in ultra-low carbon steel sample by experimental: (**a–c**), *w*(FeO)% = 1%; (**d–f**), *w*(FeO)% = 15%, R = 3; (**g–i**), *w*(FeO)% = 15%, R = 4.6.

The steel samples at all stages of the experiment were examined and analysed by Aspex. To reflect the difference in the change of characteristic inclusions in the steel, this study selected three groups of steel samples with different slag oxidation and basicity change time points for comparative analysis, as shown in Fig. 8.

Figure 8a–c shows the evolution of inclusions in steel when slag oxidation is low. As can be seen from the energy spectrum, the inclusions are mainly Al and O, without Ti. This indicates that under the action of low oxidation slag, the oxygen introduced into the steel completely combines with [Al]_s_ to form Al_2_O_3_ inclusions, while the oxidation of [Ti] is completely suppressed. From the perspective of inclusion morphology, clusters of Al_2_O_3_ inclusions form at the beginning. As the reaction time progresses, the morphology of the inclusion changes into stick-like and dendritic shapes. According to the research results of Yoon, *et al*.^25^, this morphological change is mainly due to the occurrence of supersaturated [O] or [Al]_s_ in steel. However, the [O] and [Al]_s_ in the initial liquid steel were in equilibrium. The (FeO) content in the slag was also low, with little oxygen transfer to the steel. Thus, the only source of supersaturated oxygen in the steel was the (SiO_2_) in the slag. The self-dissociation of (SiO_2_) transfers oxygen to the steel. This leads to stick-like and dendritic inclusions of Al_2_O_3_ forming in the steel. This test result also validates the conclusions predicted by the model in Fig. 6a.

Figure 8d–f shows the evolution of inclusions in steel with *w*(FeO)% = 15% slag and a basicity of 3. Figure 8 shows that the size of the inclusions in the steel increases when the oxidation of the slag increases, and the maximum size can reach 20 μm. Al_2_O_3_ inclusions are formed at the beginning of the reaction, and Ti-O inclusions begin to appear in the steel as the reaction progresses. This is mainly because the initial high concentration of oxygen rapidly combines with [Al]s in the steel to form Al_2_O_3_ inclusions. As the [Al]_s_ content in the steel decreases, the supersaturated oxygen combines with [Ti] to form Ti-O inclusions. From the point of view of inclusion morphology, the inclusions mainly present in cluster and sphere shapes when the supersaturated oxygen occurs in the steel.

Figure 8g–i shows the evolution of inclusions in steel with *w*(FeO)% = 15% slag and a basicity of 4.6. By comparing Fig. 8d–f, it can be seen that the shape and size of inclusions changed greatly. At the beginning of the reaction, the Al_2_O_3_ inclusions were approximately 20 μm. Subsequently, Al-Ti-O inclusions below 5 μm appeared, and the size of inclusions showed a downward trend. This is mainly because high basicity slag can promote the rapid removal of large particle inclusions. Moreover, Al_2_O_3_ inclusions floating in slag further improve the *w*(CaO)/*w*(Al_2_O_3_) of the slag and further improve the purity of liquid steel. This result is also consistent with the conclusion predicted by the model. The morphology of inclusions shows that Al_2_O_3_-TiO_x_ inclusions generally have a spherical shape with TiO_x_ as the nucleation centre and a size of 4–6 μm, and the edges are covered by Al_2_O_3_. According to research by Park^26^ and Doo^27^, when Ti-Fe alloy is added to steel, it will lead to locally high [Ti] or [O] concentration. Al_2_O_3_-TiO_x_ inclusions are easily formed in the locally high [Ti] concentration area. In this study, the [Ti] distribution in steel after melting with ultra-low carbon steel was relatively uniform. There was no locally high [Ti] concentration region. Therefore, the formation of Al_2_O_3_-TiO_x_ inclusions was caused by high oxidising slag rather than the local oxidation of [Ti]. After bottom blowing and standing, the composition of the liquid steel was uniform. The Al_2_O_3_-TiO_x_ inclusions decomposed and disappeared, forming stable Al_2_O_3_ and TiO_2_ inclusions. These experimental results are consistent with those predicted by the model.

## Conclusions

(1) The kinetic model developed by the Macro Processing module in the FactSage software program can predict the re-oxidation behaviour of ultra-low carbon steel via slag with different oxidisabilities. The calculated results of the model show good agreement with the experimental data from the laboratory thermal simulation. This model can predict and guide the accurate secondary oxidation control process for the actual production.

(2) During the slag re-oxidation process, when *w*(FeO)% = 1%, the oxygen that generates inclusions is mainly derived from the self-dissociation reaction of (SiO_2_) in the slag. With the increase in (FeO) content, the oxygen transfer from slag to steel is marked. The oxygen in steel mainly comes from the decomposition reaction of (FeO) in the slag. Moreover, the decomposition reaction of (FeO) suppresses the self-dissociation reaction of (SiO_2_) in the slag. In addition, the oxidation of [Ti] in steel can be suppressed by increasing the basicity and ratio of CaO to Al_2_O_3_ in the initial slag, while the number of inclusions can be reduced.

(3) When the oxygen in steel is supersaturated and the slag is low in oxidation, stick-like and dendritic inclusions of Al_2_O_3_ form easily. As the oxidation of the slag increases, the size of the inclusions increases, and it presents clusters and spheres shape. In addition, the supersaturated oxygen in steel easily forms unstable Al_2_O_3_-TiO_x_ inclusions with [Ti]. As the components in the liquid steel tend to be uniform, Al_2_O_3_-TiO_x_ inclusions decompose and disappear, forming stable Al_2_O_3_ and TiO_2_ inclusions.
